# Molecular characterization of similar Hb Lepore Boston-Washington in four Chinese families using third generation sequencing

**DOI:** 10.1038/s41598-024-60604-7

**Published:** 2024-04-30

**Authors:** Jianlong Zhuang, Na Zhang, Yu Zheng, Yuying Jiang, Yu’e Chen, Aiping Mao, Chunnuan Chen

**Affiliations:** 1 Prenatal Diagnosis Center, Quanzhou Women’s and Children’s Hospital, Quanzhou, 362000 Fujian Province People’s Republic of China; 2 Yaneng BIOscience (Shenzhen) Co. Ltd., Shenzhen, 518000 Guangdong People’s Republic of China; 3 Department of Ultrasound, Quanzhou Women’s and Children’s Hospital, Quanzhou, 362000 Fujian Province People’s Republic of China; 4 Berry Genomics Corporation, Beijing, 102200 China; 5 Department of Neurology, The Second Affiliated Hospital of Fujian Medical University, Quanzhou, 362000 Fujian Province People’s Republic of China

**Keywords:** Thalassemia, Third-generation sequencing, Gap-PCR, Sanger sequencing, Hb Lepore, Genetics, Health care

## Abstract

Hemoglobin (Hb) Lepore is a rare deletional δβ-thalassemia caused by the fusion between delta-beta genes, and cannot be identified by traditional thaltassemia gene testing technology. The aim of this study was to conduct molecular diagnosis and clinical analysis of Hb Lepore in four unrelated Chinese families using third generation sequencing. Decreased levels of mean corpuscular volume (MCV), mean corpuscular hemoglobin (MCH) and an abnormal Hb band were observed in the probands of the four families. However, no common α and β-thalassemia variants were detected in the enrolled families using polymerase chain reaction-reverse dot blot hybridization based traditional thalassemia gene testing. Further third-generation sequencing revealed similar Hb Lepore-Boston-Washington variants in all the patients, which were resulted from partial coverage of the *HBB* and *HBD* globin genes, leading to the formation of a delta-beta fusion gene. Specific gap-PCR and Sanger sequencing confirmed that all the patients carried a similar Hb Lepore-Boston-Washington heterozygote. In addition, decreased levels of MCH and Hb A2 were observed in the proband’s wife of family 2, an extremely rare variant of Hb Nanchang (GGT > AGT) (HBA2:c.46G > A) was identified by third-generation sequencing and further confirmed by Sanger sequencing. This present study was the first to report the similar Hb Lepore-Boston-Washington in Chinese population. By combining the utilization of Hb capillary electrophoresis and third-generation sequencing, the screening and diagnosis of Hb Lepore can be effectively enhanced.

## Introduction

Thalassemia is an inherited hemolytic anemia caused by the reduced or absent synthesis of one or more globin chains in hemoglobin due to globin gene deletion or mutation, it is commonly categorized into two groups: α-thalassemia and β-thalassemia^1–3^. Among them, β-thalassemia is mainly caused by point mutations in the β-globin gene, while deletional variants are rare^4,5^. Chinese ^G^γ^+^(^A^γδβ)^0^-thalassemia and SEA-HPFH are the most common δβ-thalassemia in Chinese populations caused by partial *HBB* and *HBD* deletion^6^. The Hb Lepore are a group of structural defects, which are composed of two normal α chains and two δβ fusion chains, causing by recombination events between the δ- and β-globin genes, resulting in δ and β fusion gene and δβ-thalassemia. Hb Lepore occurs worldwide, the Boston-Washington, Baltimore, Hollandia and Leidan have been described in the database and literature^7–11^.

Common α- and β-thalassemia gene variants in China are typically diagnosed by PCR-reverse dot blot hybridization (PCR-RDB) and gap-PCR^12^. However, traditional diagnostic tools may lead to misdiagnosis of rare thalassemia genotypes. At present, the third-generation sequencing (TGS) technology has been gradually used for thalassemia molecular diagnosis, with an obvious advantage in revealing globin genes single-nucleotide variants, short insertions/deletions and structural variants^13,14^. Increasing rare and novel globin gene variants have been identified using long-read sequencing^13–16^.

In this study, four families with abnormal hematological screening results indicating of rare thalassemia carriers underwent further investigation using third-generation sequencing. All of the proband’s in these families harbored Hb Leprore variants, which were resulted from partial coverage of the *HBB* and *HBD* globin genes, leading to the formation of a delta-beta fusion gene. Moreover, our findings could provide valuable reference in conducting TGS for rare and novel thalassemia diagnosis.

## Materials and methods

### Subjects

Four unrelated families from the Quanzhou region in Southeast China, with abnormal routine blood analysis and Hb capillary electrophoresis screening results, were enrolled in this study. All the probands had similar abnormal Hb Lepore bands located in Hb zone 6, manifested decreased levels of MCV and MCH, and suspected as thalassemia carriers. All the enrolled members in the four families denied recent blood transfusions. After written informed consents were signed, peripheral blood samples were collected for further molecular diagnosis in the enrolled subjects. This study was approved by the ethics committee of The Women’s and Children’s Hospital of Quanzhou (2021No.61).

### Hematological screening

Routine blood analysis for mean corpuscular volume (MCV) and mean corpuscular hemoglobin (MCH) levels detection was conducted using an automated cell counter (Sysmex XS-1000i; Sysmex Co., Ltd., Kobe, Japan). Hb capillary electrophoresis (Sebia, Evry Cedex, France) was performed to detect the levels of Hb A, Hb A2 and Hb F and other abnormal hemoglobin bands. Positive hematological screening results included at least one of the following criteria: MCV < 82 fL, MCH < 27 pg, Hb A2 > 3.4%, Hb A2 < 2.6%, Hb F > 2.0% and abnormal Hb bands.

### Traditional thalassemia gene testing

All of the members in the four families did further thalassemia gene testing. Genomic DNA of the members in the enrolled families were extracted using an automatic nucleic acid extractor (Ruibao Biological Co., Ltd). Twenty-three types of common α-thalassemia and β-thalassemia variants in the Chinese populations were detected through PCR-RDB according to the manufacturer’s protocol (Yaneng Biological technology Co., Ltd., Shenzhen)^17^.

### Third-generation sequencing

The third-generation sequencing based on PacBio Sequel II platform for globin genes variants detection was conducted as the description of our previous study^16^. Genomics DNA were extracted and then sent to Berry Genomics laboratory for third-generation sequencing. Briefly, the purified DNA samples were quantified using the Qubit dsDNA BR assay kit (ThermoFisher Scientific). Then, optimized primers were used to generate specific amplicons that encapsulated known structural variation regions, as well as single nucleotide variations in the *HBA1/2* and *HBB* globin genes, based on databases such as HbVar, Ithanet, LOVD, and LOVD-China. After purification and end repair, double barcode adaptors were ligated to the 5’ and 3’ ends and Sequel Binding and Internal Ctrl Kit 3.0 (PacBio) was used to prepare SMRT bell libraries. Finally, third-generation sequencing was performed on the PacBio Sequel II System after primed DNA-polymerase complexes were loaded onto the SMRT cells^16^.

Following alignment of the subreads, the consensus circular sequence was mapped to the GRCh38 reference and variants were called (FreeBayes software, version 1.2.0). Cis- and trans-configuration between two variants in the long reads was analyzed using WhatsHap (version 0.18) software. The alignments of variant and wild-type molecules were visualized using the Integrative Genomics Viewer^16^.

### Specific gap-PCR amplification and Sanger sequencing

Specific gap-PCR or Sanger sequencing were used to confirm the rare or novel globin gene variants detected by third-generation sequencing. Gap-PCR was used to identify the deletion breakpoints. We designed specific primers according to the known DNA sequences around the breakpoints or the globin gene sequence variants. The primers sequences were as followed, P1: AGAGATGCGGTGGGGAGATA and P2: AACGATCCTGAGACTTCCACA. All primers were synthesized at Sangon Biotech (Shanghai). Gap-PCR reaction system: 5 × buffer 5 μL, 25 mmol dNTPs 0.2 μL, 25 mmol MgCl_2_ 1.5 μL, Taq enzyme 2.5U, 10 μmol primers 1 μL each, template 2 μL, and plus ultra-pure water to 25 μL. The amplification conditions were 95 °C for 10 min, then 35 cycles of 94 °C for 1 min, 62 °C for 30 s, 72 °C for 1 min, and finally 72 °C for 5 min. Electrophoresis analysis was performed and then the purified electrophoresis products were sent for Sanger sequencing. In addition, Sanger sequencing was also performed for globin gene variants. The sequenced data were analyzed with GenBank NG_000007.3 as their reference sequences.

### Ethics approval and consent to participate

This study was approved by the ethics committee of The Women’s and Children’s Hospital of Quanzhou (2021No.61). We received informed consent from the study participants and their parents, and they agreed to the publication of a report on the study. All procedures performed involving human participants were in accordance with the ethical standards of the institutional and/or national research committee and with the 1964 Helsinki declaration and its later amendments or comparable ethical standards.

## Results

### Hematological screening results

**Figure 1:**
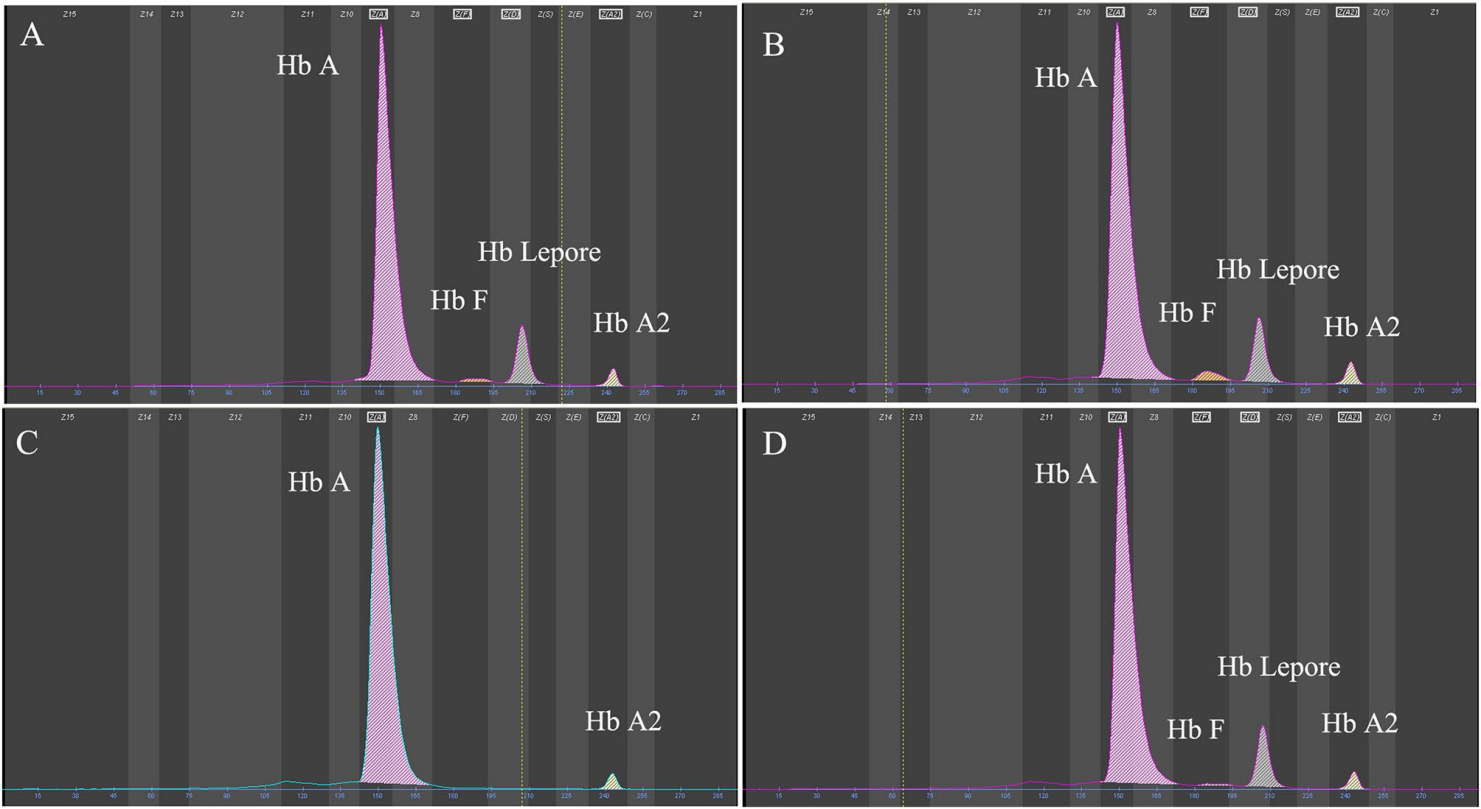
Hemoglobin capillary electrophoresis results of the enrolled families. (**A**,**B**,**D**) All of the probands in family 1, 2, and 4, elicited abnormal Hb bands in zone 6, which indicated existence of Hb variants. (**C**) Hemoglobin capillary electrophoresis results revealed a decreased level of Hb A2 in proband’s wife of family 2.

The hematological screening results of the enrolled four families were described in Table 1. All of the members in four families had decreased levels of MCV and MCH detected by routine blood analysis. As elicited in Table 1 and Fig. 1, subsequent Hb capillary electrophoresis results demonstrated similar four Hb bands in the four probands of the families as well as the proband’s daughter in family 1, including Hb A, Hb A2, Hb F, and Hb zone 6 bands. Among these five subjects, the abnormal Hb zone 6 (Hb Lepore) levels was obvious range from 7.3 to 10.5%, and Hb F levels range from 0.7 to 14.1%. In addition, both of the proband’s wife in family 2 and family 3 only had a decreased level of Hb A2, who were suspected as α-thalassemia carriers.

**Table 1:** The hematological and molecular analysis of the enrolled families.

	Family 1		Family 2		Family 3		Family 4
	Proband	Daughter	Proband	Wife	Proband	Wife	Proband
Sex-age	F-37	F-2	M-29	F-28	M-31	F-28	M-27
RBC(10^12^/L)	4.73	5.10	7.31	5.15	5.04	4.53	6.19
Hb(g/L)	108	112	146	138	97	124	150
MCV(fl)	70	66.7	63.7	84.3	69.1	80.4	65
MCH(pg)	22.8	21.7	20	26.8	22.8	28	20.9
Hb A(%)	85.4	76.1	84.4	97.8	83.2	97.5	86.6
Hb A2(%)	2.5	2.5	2.9	2.2	2.3	2.5	2.3
Hb F(%)	1.6	14.1	2.5	0	5.1	0	0.7
Hb zone 6(%)	10.5	7.3	10.2	0	9.4	0	10.4
α-thalassemia	αα/αα	αα/αα	αα/αα	αα^HBA2:c.46G>A^/αα	αα/αα	αα/αα	αα/αα
β-thalassemia	Hb Lepore-BW	Hb Lepore-BW	Hb Lepore-BW	β^N^/β^N^	Hb Lepore-BW	β^N^/β^N^	Hb Lepore-BW

M, Male; F, Female; N, Normal; Hb, Hemoglobin; BW, Boston-Washington.

### Traditional thalassemia gene testing results

Firstly, 23 common α-thalassemia and β-thalassemia variants in Chinese populations were investigated using PCR-RDB technique in the four families. As delineated in Table 1**,** no common α-thalassemia and β-thalassemia variants was detected. Given that the positive hematological screening results in the enrolled subjects, rare or novel globin genes variants were suspected and all of them were subject to further genetic diagnosis.

### Third-generation sequencing results

**Figure 2:**
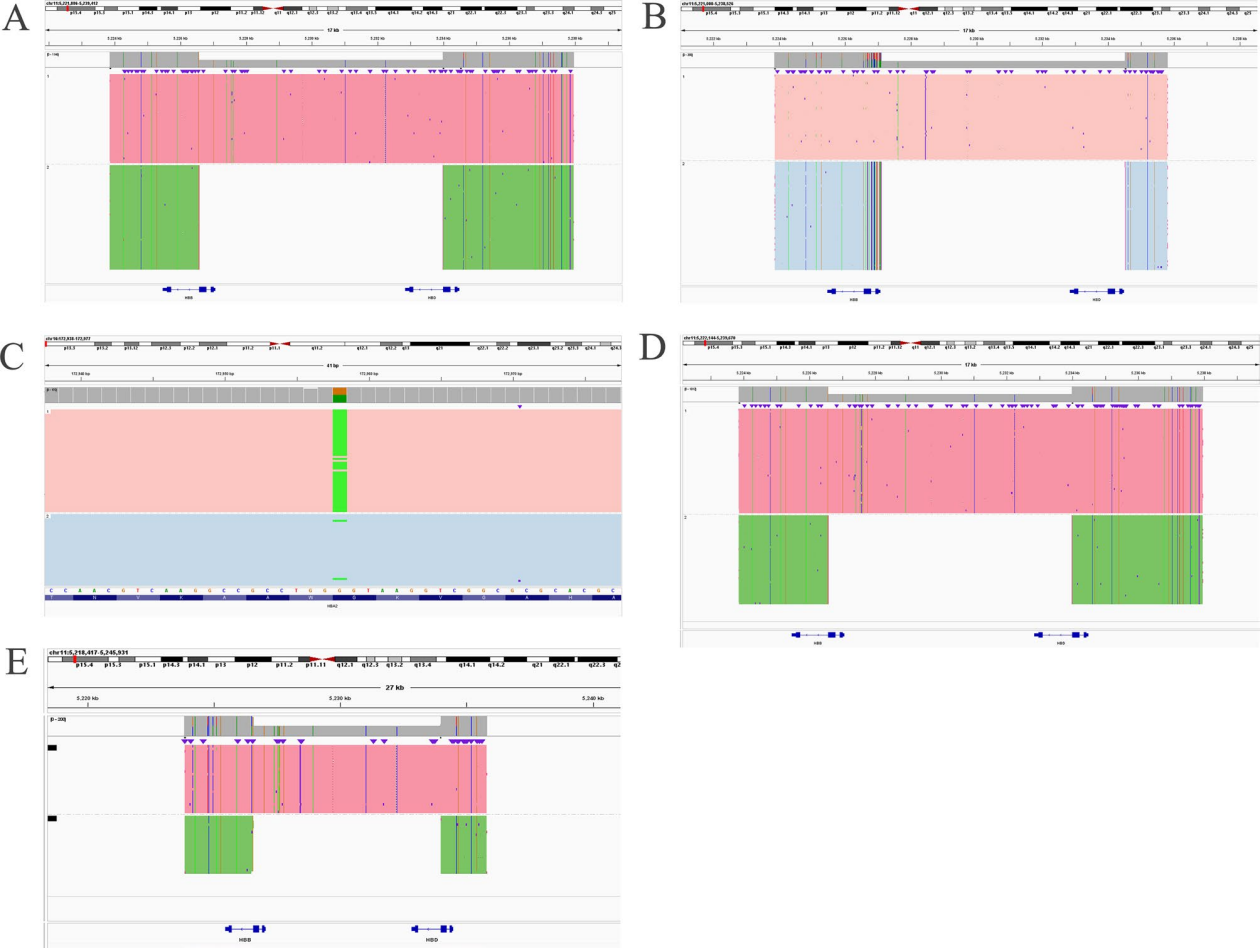
Third-generation sequencing (TGS) results of the enrolled families. (**A**) In the probands of family 1, a 7.414 kb deletion (NG_000007.3:g.63633_71046del) that partially covering *HBB* and *HBD* globin genes was identified. (**B**) In the proband of family 2, a 7.415 kb deletion (NG_000007.3:g.63632_71046del) in *HBB* and *HBD* globin genes was detected using TGS. (**C**) A variant of Hb Nanchang (GGT > AGT) (HBA2:c.46G > A) was identified in the proband’s wife of family 2. (**D**) A 7.414 kb deletion (NG_000007.3:g.63633_71046del) that partially covering *HBB* and *HBD* globin genes was identified in family 3. (**E**) A 7.415 kb deletion (NG_000007.3:g.63626_71040del) was identified in the proband of family 4.

In order to further investigate the thalassemia genotype of the suspected families, TGS based on single-molecule real time sequencing was performed for globin genes detection including single nucleotide variation and structure variants. As depicted in Table 2 and Fig. 2, TGS detection revealed large deletions that partially encompassed the *HBB* and *HBD* globin genes in four probands from the families, which located in the region of Hb Lepore-Boston-Washington. In family 1, the proband’s daughter who exhibit similar hematological screening results also had the same deletion. In addition, a rare variant of Hb Nanchang (GGT > AGT) (HBA2:c.46G > A) at codon 15 in α2 (Fig. 2) was identified in the proband’s wife of family 2 with decreased level of Hb A2 and slightly low level of MCH. However, no relative globin genes variants was observed in the proband’s wife of family 3 who also had decreased level of Hb A2.

**Table 2:** *HBB* and *HBD* deletions detected by third-generation sequencing (TGS) and verified by Sanger sequencing.

Families	TGS (NG_000007.3)	Sanger sequencing (NG_000007.3)
Family 1	63633_71046del	63633_71046del
Family 2	63632_71046del	63633_71046del
Family 3	63633_71046del	63633_71046del
Family 4	63626_71040del	63633_71046del

### Specific gap-PCR amplification verification results

**Figure 3:**
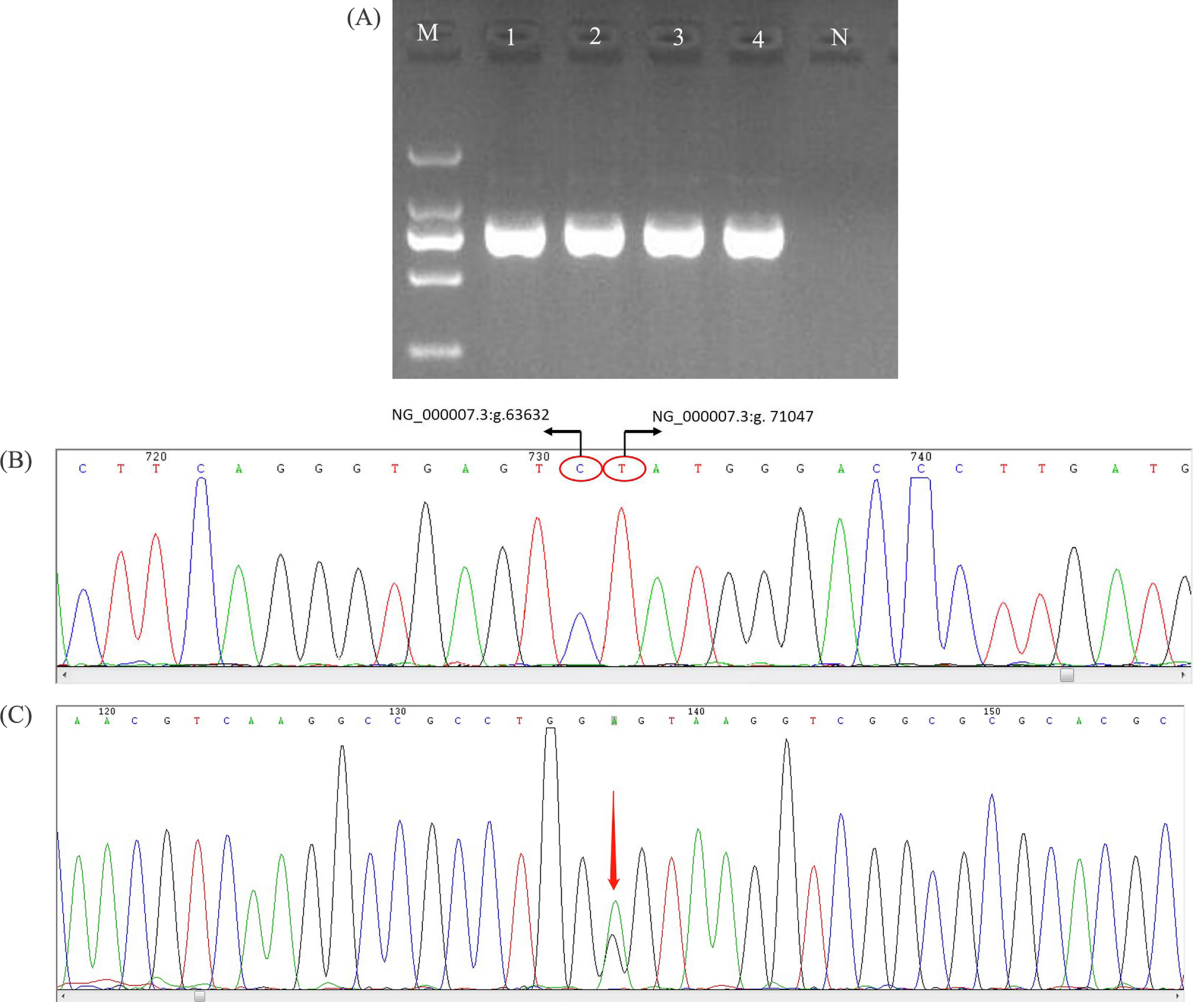
Specific gap-PCR amplification and Sanger sequencing results in the four enrolled families. (**A**) 1: family 1. 2: family 2. 3: family 3. 4: family 4. N: Normal control. (**B**) Sanger sequencing results showed that the deletion fragments of fracture in the four families was range from 63,633–71,046 bp, which was similar to Hb Lepore-Boston-Washington. (**C**) The Hb Nanchang (GGT > AGT) (HBA2:c.46G > A) variant in the proband’s wife of family 2 was verified by Sanger sequencing.

In order to confirm the deletions in the enrolled families, the specific gap-PCR amplification was further conducted. Subsequently, two specific primers were designed to amplify the breakpoints using gap-PCR technique. The gap-PCR detection results demonstrated a large deletion covering the *HBB* and *HBD* globin gene cluster in all the proband’s of the four families. Subsequently**,** the electrophoretic results delineated a 800 bp PCR product in the four families, which amplified by the primers P1 and P2 (Fig. 3).

### Sanger sequencing results

To further verify the specific location of the breakpoints in the four enrolled families, Sanger sequencing was conducted using the specific products that amplified by gap-PCR. As shown in Table 2 and Fig. 3, after alignment between sequencing results and NG_000007.3 sequence through BLAST analysis, all the patients in the four families had a partial *HBB* and *HBD* deletion fragment range from 63,633–71,046 bp, with a fragment length of 7.414-kb (NG_000007.3:g.63633_71046 del), which was almost the same with Hb Lepore-Boston-Washington (NG_000007.3:g.63632_71046 del). In addition, the Hb Nanchang (GGT > AGT) (*HBA2*:c.46G > A) identified in proband’s wife in family 2 was further confirmed by Sanger sequencing (Fig. 3).

## Discussion

Thalassemia is a hereditary blood disorder caused by globin gene synthesis disorders^4^, which included α, β, γ, δβ-thalassemias. Thalassemia is highly prevalent in South China, especially in Guangdong, Guangxi, and Hainan provinces^18–20^. Quanzhou region of Fujian province, located in Southeast China, displays a high prevalence of thalassemia, with a diversity and complexity of thalassemia^21–23^. Common α- and β-thalassemia gene variants in China are typically diagnosed by PCR-RDB and gap-PCR^12^, but they have limitations in the diagnosis of rare thalassemia variants. Third-generation sequencing technology has significant advantages in the diagnosis of thalassemia, and can detect almost all known globin gene variants^13,14^. In our study, third generation sequencing was performed to investigate globin gene variants in four Chinese families with abnormal Hb variants, all of the suspected members harbored a large 7.414-kb deletion that partially affected the *HBB* and *HBD* globin genes (NG_000007.3:g.63633_71046 del), which closely resembled the Hb Lepore-Boston-Washington variant (NG_000007.3:g.63632_71046 del).

δβ-thalassemia is a rare condition, which is caused by partial deletions in *HBB* and *HBD* globin genes. In China, the ^G^γ^+^(^A^γδβ)^0^ and SEA-HPFH genotypes were the two most common δβ-thalassemia variants^6^. In addition, our previous study indicated a higher prevalence of SEA-HPFH of δβ-thalassemia in Quanzhou region, while, Hb Lepore had not been identified^22,23^. The Lepore hemoglobins are a group of δβ-thalassemia, which is more frequently identified in Southern Europeans^11^, Caucasians in Central Portugal and in the Spanish Alta Extremadura^24^. The Hb Lepore Boston-Washington, Baltimore, Hollandia and Leidan are commonly described in the database and literature, with different recombination crossover breakpoints^7–10^. Moreover, several novel Hb Lepore variants have been identified, including Hb Lepore-Hongkong and Hb Lepore-ARUP^25,26^.

Hb Lepore has been rarely reported in Chinese populations, while, the Hb Lepore-Boston-Washington seem more prevalent in China^27^. Hb Lepore cannot be diagnosed through routine thalassemia gene testing based on PCR-RDB technology, but it can be well indicated through hemoglobin electrophoresis or high performance liquid chromatography (HPLC) technology. Heterozygotes of Hb Lepore commonly exhibit a mild hypochromic microcytic anaemia with 10–15% Hb Lepore and a slightly increased level of Hb F^7^. However, a novel Hb Lepore-Hong Kong was identified with increased level of Hb A2 and Hb F, but without a Hb Lepore band, which may due to the delta-beta fusion gene of Hb Lepore-Hong Kong that sharing the same coding sequences as *HBB*^25^. In the present study, five members in four Chinese families had decreased levels of MCV and MCH, with an abnormal Hb band in zone 6 (7.3–10.5%), and various Hb F levels (0–5.1%), which was consistent with the previous reports. Finally, all of the suspected members were identified carrying a same 7.414-kb deletion (NG_000007.3:g.63633_71046 del) in *HBB* and *HBD* globin genes, which manifest one bp difference with Hb Lepore-Boston-Washington, and we proposed to classify them as Hb Lepore-Boston-Washington heterozygosity carriers.

Hb Lepore is associated with a β-thalassemia phenotype, thus, individuals with homozygous or compound heterozygosity of Hb Lepore(s) would lead to β-thalassemia intermedia or major^28^. In this study of family 2 and family 3, the probands’ wife were also subject to TGS detection, while, no mutations in β-globin gene was observed. Interestingly, a rare Hb Nanchang (GGT > AGT) (*HBA2*:c.46G > A) variant at codon 15 in *HBA2* globin gene was identified in the proband’s wife of family 2. The *HBA2*:c.46G > A variant, which involved a GGT → AGT change and resulting in a Gly → Ser replacement at CD15 was first identified in a girl with normal hematological parameters and classified as silent α-thalassemia^29^. Subsequently, the variant of Hb Nanchang (*HBA2*:c.46G > A) was reported by Xu et al.^30^ in a Chinese population with normal hematological parameters. Notably, in our present study, the patient who harbored Hb Nanchang (GGT > AGT) (*HBA2*:c.46G > A) variant exhibited a slightly low level of MCH (26.8 pg), which may causing α^+^-thalassemia (silent α-thalassemia). In addition, as elicited in the IthaGenes database (https://www.ithanet.eu/), *HBA2*:c.46G > A was indicated to result in α^+^-thalassemia.

To summarize, our study first described the similar Hb Lepore (NG_000007.3:g.63633_71046del) in Chinese population. In addition, Hb Nanchang variants was identified in Fujian province for the first time. Our findings suggest that combining the use of Hb capillary electrophoresis and third-generation sequencing would effectively screen and diagnose Hb Lepore.

## Data Availability

The raw sequence data reported in this study have been deposited in the Genome Sequence Archive (Genomics, Proteomics & Bioinformatics 2021) in National Genomics Data Center (Nucleic Acids Res 2022), China National Center for Bioinformation/Beijing Institute of Genomics, Chinese Academy of Sciences (GSA: https://ngdc.cncb.ac.cn/gsa-human, accession number: HRA006741).
